# Why is it so hard to do co-created citizen social science? Reflecting on challenges and potential solutions

**DOI:** 10.1177/09636625261426841

**Published:** 2026-03-27

**Authors:** Jörg Matthes, Isabelle Freiling

**Affiliations:** 1 University of Vienna, Austria; 2 University of Utah, USA

**Keywords:** challenges, citizen science, citizen social science, co-creation, collaboration, research process

## Abstract

Citizen science (CS), which integrates the public into research processes, has been shown to enhance public understanding of science. Consequently, CS is increasingly employed across various disciplines, including the social sciences. In citizen social science (CSS) projects, citizen involvement typically extends beyond data collection, encompassing multiple stages of the research process—from conceptualization to data analysis. Despite the growing scholarly enthusiasm for CSS, this essay highlights key methodological and practical challenges associated with such co-created CSS initiatives. Using a systematic review of recent literature, we identify challenges across all consecutive steps of the research process involving co-creation. We conclude by advocating for a more nuanced perspective on CSS, emphasizing the need for a heightened awareness of these challenges.

Citizen science (CS) is a scientific approach in which volunteers (remunerated or not) are integrated into the research process and take roles that have traditionally been filled by academics (Bonney et al., 2009). CS is particularly common in the life, environmental, or biological sciences (Heiss and Matthes, 2017), but also increasingly in the social sciences, where it is called *Citizen Social Science* (CSS; see Freiling and Matthes, 2024; Tauginienė et al., 2020; Thomas et al., 2021). CS can be classified into different types of projects depending on their level of citizen involvement. In *contributory* projects, citizens are mainly involved in data collection (Bonney et al., 2009). In *collaborative* projects, citizens’ involvement is a bit higher, with citizens not only collecting data, but also helping scientists to refine the design, analyze the data, and distribute the results. In *co-created* projects, citizens are involved in every project phase, thus, already in the phase of establishing what the project will be about. Such projects have also been called “extreme citizen science” (English et al., 2018; Kwon et al., 2022).

Overall, CS is met with a great deal of enthusiasm (e.g. Albert et al., 2021; Thomas et al., 2021; Van Oudheusden et al., 2024): It can democratize science, enable significant knowledge gains, or increase the public understanding of science (Van Oudheusden et al., 2024). At the same time, scholars have also questioned the value of CS (e.g. Davis et al., 2023; English et al., 2018). In particular, in a meta-analysis of 895 citizen science projects, Davis et al. (2023) found that three-quarters of all CS projects did not produce a single peer-reviewed paper, with an average time from project launch to publication of more than 9 years. The authors conclude that “the ‘science’ element of most citizen science projects is largely irrelevant as it is never validated or communicated” (p. 1). Furthermore, they suggest that co-created CS projects may be called “citizen engagement projects” rather than “citizen science.” Their argument is that the value of such projects may not lie in doing actual science, but rather in citizen engagement and democratization as well as citizen involvement.

In this essay, we follow up on the findings by Davis et al. (2023). While we do not argue to take the “science” out of the term “citizen science” for co-created projects, we discuss potential reasons for the findings reported by Davis et al. (2023). Thus, we ask, *Why is it so hard to do co-created CS*? In particular, we focus on CSS. In CSS, citizens are typically more than data collectors; projects are at least collaborative, if not fully co-created (e.g. Albert et al., 2021; for a critical view, see Freiling and Matthes, 2024; Van Oudheusden et al., 2024). Unlike natural sciences, which often focus on physical phenomena, CSS emphasizes human behavior and social interactions, introducing unique practical challenges for co-creation. In this essay, we systematically analyze the recent literature to discuss the challenges of co-creation in CSS across all stages of the research process. We advocate for a more thorough consideration of the practical and methodological challenges inherent in conducting CSS.

## 1. Challenges in co-created CSS

Previous discussions of challenges either focus on one outcome of CS projects like learning and education (e.g. National Academies of Sciences, Engineering, and Medicine, 2018; Roche et al., 2020), or they discuss selected aspects (e.g. Palacin et al., 2021; Senabre et al., 2018). To the best of our knowledge, there is no systematic discussion about the challenges of co-creation in the context of CSS. According to scholars (Haklay, 2013; Kwon et al., 2022), co-creation is meant to occur during all stages of the research process, that is, problem definition, theory development, methods and data collection, data analysis and interpretation, scientific and public dissemination, and evaluation. We do not focus on general challenges such as citizen motivation and ethical questions, which have been discussed at great length in previous work (e.g. Ozolinčiūtė et al., 2022) and which do not specifically refer to co-created CSS but to CS in general.

We base this discussion on a brief systematic analysis of the recent literature (2015–2025). Following the guidelines of Preferred Reporting Items for Systematic Reviews and Meta-Analyses (PRISMA) (Page et al., 2021), we conducted a literature search in the Web of Science using the terms “citizen science” AND “social science” AND “challenges OR limitations OR problems OR risk OR limitation.” These keywords had to appear in the title or abstract. The search was conducted on April 9, 2025 and yielded *N* = 258 records. Irrelevant papers not in line with the selection criteria were screened out (i.e. not social science and/or no challenges addressed). The remaining *n* = 19 papers were assessed qualitatively to identify whether they mentioned or discussed the challenges along the consecutive steps of the research process. In what follows, we present a discussion of those challenges based on this analysis. The full search string, as well as a list of the final 19 papers, can be found on OSF at https://osf.io/uyzw2/overview?view_only=006830fd8a5a44c0ae034d610d5e18a8.

### Problem definition

The identified papers touch upon the question of how a research problem underlying a research topic is identified (Kwon et al., 2022). Following the definition of CSS as having citizen involvement at all stages of the research process, citizens should be involved in the problem definition as well. In most CSS research, however, topics are suggested by researchers rather than co-created with citizen scientists. For instance, in a CSS study that examined how citizens could be mobilized to collect empty housing data, the topic and problem definitions were provided by the researcher (see Albert, 2021). Researchers and citizens can have different perceptions of what is relevant. For instance, as noted by Froeling et al. (2024), citizens prefer research questions based on pre-existing interests rather than an analysis of the literature, while researchers may think more of relevance for advancing scientific knowledge in their discipline. Also, it appears rather challenging to discuss the selection of a problem on equal footing, which is, however, what the notion of co-created CS suggests. Ultimately, scientists may sit in the driver’s seat to determine what a good and valuable scientific problem or topic is, and they likely have to decline working on a problem or topic that is non-scientific within their job. For instance, in a study by Froeling et al. (2024), researchers predetermined the overall research themes, but citizens defined a specific research question within those themes. Thus, a hierarchy was still maintained. Likewise, while (Thomas et al., 2021) pointed to the importance of scientists acting as workshop moderators to minimize inequalities, they also acknowledged that moderation may still be, what they call, an instrument of domination.

Obviously, it can be challenging to involve citizen scientists at such an early stage of the project as the problem definition. In case of third-party funding, the topic needs to be determined before the start of the project, that is, potentially before citizen scientists are involved. This results in citizen scientists only being able to co-create the specific sub-aspects of a topic (see Froeling et al., 2024). Also, in most countries, CS is not institutionalized (except for science museums, see Méndez and Cortés-Fossati, 2021). As a consequence, even if citizens want to suggest a problem definition, there is often no particular place to turn to.

### Theory development

Scholars also noted the issue of theory development (Froeling et al., 2024; Pykett et al., 2020). In the social sciences, theories often involve abstract concepts (Heiss and Matthes, 2017). To participate in theory work, citizens must be able to define and understand these concepts. Yet citizen scientists’ lack of scientific knowledge is a well-known problem (e.g. Martin et al., 2016) regarding working on equal footing in CS. Contrary to citizen scientists, professional researchers have undergone a lengthy learning process in their field. Learning the required skills for selecting relevant theories and applying them to a specific research project takes time and practice (National Academies of Sciences, Engineering, and Medicine, 2018; National Research Council, 2007). At universities, we teach students about theories, often starting with an introductory course, possibly followed by topical courses focusing on some sub-fields, typically spanning over several years. In most cases, citizen scientists therefore lack knowledge of basic theories or facts relevant to the CSS project’s topic, making it difficult for citizen scientists to equally contribute to the project (see Heiss and Matthes, 2017).

For instance, in the context of stress research, Pykett et al. (2020) have pointed to challenges related to defining the most basic concepts, such as stress, the body, physiology, and emotions. Froeling et al. (2024) encountered challenges when “engaging effectively with citizens throughout the study, harmonizing citizens’ knowledge and values with the academics’ expertise, managing civic expectations, making complex concepts understandable to citizens” (p. 1.). In line with this, Göbel et al. (2022) found that people from the educated, employed middle class are more likely to participate as compared to other groups. This underlines findings of earlier research showing that citizen scientists who lack education face more difficulties to contribute (Martin et al., 2016).

### Methods and data collection

Concerns about the data quality of CS projects are not new (Aceves-Bueno et al., 2017; Balázs et al., 2021; Heiss and Matthes, 2017), and specific recommendations have been developed (Balázs et al., 2021; English et al., 2018). It is thus not surprising that this issue was also touched upon in the sampled papers (e.g. O’Grady and Mangina, 2024; Thomas et al., 2021). In the social sciences, data collection may go beyond the notion of contributory CS and, for instance, involve qualitative methods that citizen scientists are involved in (e.g. developing an interview scheme). In such co-created CSS projects, there are several additional challenges. As with theory, doing CSS requires basic knowledge about research methodology, which may be difficult to acquire. Also, in contrast to natural sciences, most social scientists are interested in latent rather than directly observable constructs. Measurement of those latent constructs is far less standardized and the criteria for what constitutes “good” data are not as clear for CSS (Balázs et al., 2021). An example of what CSS often measures is human behavior. Human behavior is more difficult to observe than the objects of observation in natural sciences. If observation of constructs is more subjective (e.g. reporting an impression) and not standardized, as compared to strictly objective (e.g. measuring water quality), individual perception and observation biases need to be considered (Heiss and Matthes, 2017). For instance, Thomas et al. (2021) found that citizen scientists faced difficulties in suspending their personal positions and opinions. Of course, there can be training, but such training needs to be extensive. O’Grady and Mangina (2024) conducted a survey with citizen scientists and found that, while formal and informal training were offered, the participation in those trainings was alarmingly low (less than 50%). If training was offered, it only focused on data collection and analysis, but not on other standard topics such as research ethics or transparent reporting.

Several scholars still acknowledge the need for training citizen scientists (e.g. Jallad et al., 2021; Mintchev et al., 2024), while acknowledging at the same time that such training “may not turn people into professional academics—as is the case with trainings in university PhD programs, for example—but to enable citizen scientists to integrate the experience and knowledge they already have into new initiatives that use research as a pathway to social change” (Mintchev et al., 2024: 497). For example, as a solution to the challenge of only less than half of citizen scientists participating in training—if it was offered in the first place—Thomas et al. (2021) asked citizen scientists to learn what they called “low-threshold research methods,” rather than the real scientific methods that require substantial amounts of training.

It follows that CSS is facing a dilemma when it comes to methods: on the one hand, overly complex social science methods may be unattractive to citizen scientists, potentially dampening the motivation to participate. On the other hand, overly simplified methods may compromise data quality (Aceves-Bueno et al., 2017; Balázs et al., 2021; Heiss and Matthes, 2017; Thomas et al., 2021).

### Data analysis and interpretation

There are also considerable challenges when it comes to data analysis and visualization, mostly related to scientific rigor (English et al., 2018). Similar to data collection, data analysis and interpretation often require specialized skills, necessitating heavy training. Especially for advanced data analysis techniques, it may be difficult for citizens to acquire skills in the analytical steps or the necessary software. Again, as for data collection, especially since social sciences study human behavior, citizens also need to be trained to approach the analysis and interpretation of data in an intersubjective way that approaches objectivity as closely as possible. Besides skills, citizen motivation is a crucial factor. While citizens may be interested in the substantive research question or phenomenon, they may find data analytical, methodological, or statistical aspects to be less rewarding. When it comes to data interpretation, citizen scientists’ perspectives may be very helpful, as they can offer inside knowledge that may be inaccessible to professional scientists. Some challenges may nevertheless arise, such as the correct interpretation of statistical information in quantitative research (Bonney et al., 2009; Shirk et al., 2012). In qualitative research, data interpretation is closely tied to an in-depth examination of the material, which requires considerable amounts of time and resources (i.e. “crawling through the data;” see Guthrie, 2010).

### Scientific and public dissemination

Only a few papers explicitly address challenges related to the dissemination of research findings. Hinojosa et al. (2021) acknowledged that involvement in writing papers for submission to journals was uncommon for citizen scientists, and if it happened, it had to be calibrated to the levels of knowledge of the citizen scientists. In fact, scientific dissemination is likely to be challenging. Lewis (2022) noted structural barriers of journal publication systems as well as publication fees and time costs associated with dissemination. Typically, scientific publications require specific skills and knowledge, for instance, about academic language or responding to reviewers. While scientists are, or should be, intrinsically and extrinsically motivated to publish in scientific journals or books, the motivation on the side of the citizen scientists may be smaller. Also, papers may be rejected, revised, and submitted to another outlet, which can be time-intensive. In some cases, this process can span over years, which may be discouraging to citizens. Mintchev et al. (2024) have noted that long-term commitments of citizen scientists are particularly important.

In non-scientific dissemination, it is conceivable that citizens are interested in participating in public events or talking to journalists. However, communicating research findings to the public can lead to controversy or negative public reactions. Unlike scientists, citizens receive no formal training or protection by a research institution, nor do they have access to psychological counseling, legal advice, or similar services present at universities.

### Project evaluation

Evaluation is a key step in CS research, meaning that in co-created projects, citizens are ideally also involved in the evaluation. Such co-evaluation ensures that evaluation criteria are citizen-driven, rather than entirely researcher-driven, and that citizens are included in finding solutions for problems that may occur in a (process) evaluation. While challenges with respect to evaluation were not explicitly discussed, they may arise due to constraints in time and skills, as argued above. Whereas citizens may be interested in the substantial research question or research object, they may be less interested in the evaluation. Especially when it comes to impact evaluation, some outcomes can often only be observed in the long run, several years after the project, requiring resources. Against this background, involving citizen scientists in an additional role seems highly ambitious, to say the least. Finally, evaluations typically include pre-post comparisons, qualitative or quantitative. This necessitates fixed evaluation criteria before the start—or at the very beginning—of a project. It follows that citizen scientists would need to be involved even before the actual project starts. This may be the case in bottom-up CS projects (i.e. projects developed by citizens), but not in top-down projects (i.e. projects suggested by scientists).

## 2. Potential solutions

Since the challenges that CSS is facing are not insurmountable, we offer some recommendations (Table 1; see also English et al., 2018; Hollis et al., 2022; Mintchev et al., 2024). Since different contexts and topics may require different kinds of solutions, we do not believe these solutions may fit every CSS project. By and large, however, we believe they will be helpful. Starting with the problem definition, we suggest that research institutions establish CS centers, such as the UCL Citizen Science Academy, where interested citizens can suggest their own research topics, which are then picked up or discussed by professional researchers. Potential gaps in what defines a problem between citizens and researchers remain a challenge. Ideally, taking the notion of co-created CSS seriously, citizens should be involved in grant acquisition endeavors as well. So far, to the best of our knowledge, there are hardly any funding schemes that explicitly call for citizen involvement in the writing of grant applications. Funding agencies could change this in their calls by requiring citizen involvement early on, that is, when a grant application is prepared.

**Table 1.** Challenges and potential solutions.

Challenges	Potential Solutions
Problem definition: *Involving citizen scientists at an early stage is unrealistic, given a lack of institutionalized CS*	• Establishment of institutionalized CSS centers • Funding schemes targeting citizens
Theory development: *There is an unequal knowledge of theories between citizen scientists and professional researchers*	• ‘Textbooks’ for citizens • Science communication specifically targeting citizen scientists
Methods and data collection: *Data quality is difficult to secure, particularly for non-standardized measurements*	• Workshops, trainings, and video tutorials • Strict data quality monitoring • Development of best practice guidelines
Data analysis and interpretation: *Citizen scientists may lack skills and motivation to do advanced data analyses*	• Workshops, trainings, and video tutorials • Fair remuneration
Scientific and public dissemination: *Citizen scientists may lack skills and time for the dissemination process*	• Workshops, trainings, and video tutorials • Providing support for negative consequences of outreach that universities provide to their employees • Co-authorship and fair remuneration
Project evaluation: *Evaluation is often detached from the research question and, therefore, less appealing to citizen scientists*	• Recruitment of additional citizen scientists for evaluation • Additional remuneration for evaluation

With respect to theory development, we suggest a formalized introduction of citizen scientists in CSS projects to major social science theories. These could be provided, for instance, by popular textbooks targeting citizen scientists or by science communication efforts that specifically focus on identifying relevant theories to bridge the gap between professional researchers and citizen scientists.

Data quality has long been an issue in CS research (Aceves-Bueno et al., 2017; Balázs et al., 2021; Heiss and Matthes, 2017). Here, workshops, videos, or tutorials may be needed to train citizen scientists to control these biases. We also need to develop best-practice guidelines for data collection and a systematic approach to quality control for social science data. A similar challenge was identified for data analysis and interpretation, where it may be difficult for citizens to acquire the necessary skills. Again, trainings and video tutorials may be one solution, and best-practice recommendations and guidelines need to be developed. Fair remuneration may help motivate citizens to acquire the necessary skills, for instance, by paying citizen scientists for their time when they participate in those trainings.

Regarding scientific and public dissemination, citizen scientists need to be well-prepared for this role, for instance, through trainings and tutorials, and they need to be included as co-authors in scientific publications given their substantial contributions to knowledge generation, which perhaps challenges existing publication cultures. Citizen scientists should also be provided with access to psychological counseling, legal advice, or similar services present at universities, to reduce negative consequences that outreach can have.

The evaluation of the CSS project needs to be made attractive to citizen scientists. Perhaps it is necessary to involve additional citizen scientists, primarily for the purposes of evaluation, to avoid overburdening citizen scientists. Of course, these additional citizen scientists need to be remunerated as well.

## 3. A modest view

Overall, we call for a more modest view on CSS. This involves a more careful weighing of the practical and methodological challenges against the prospects and gains of doing CSS. Not all social science topics may be equally suited for a CSS perspective, and not everything that has been called CSS is actually CS, or “good” CSS. Clearly, CSS should be a means to an end, which is generating genuine scientific knowledge that is otherwise not attainable. It should not be an end in itself that is pursued primarily on normative grounds, without critically evaluating the generated knowledge gains.

Furthermore, CSS should move beyond the case-study level, with limited theoretical and practical generalizability. More efforts are needed to establish institutionalized CS, rather than “pop-up”-CS for single projects. That is, we need to build structures for long-standing citizen involvement, and spaces for citizens to develop and co-create their ideas, such as interdisciplinary centers for CS and CSS.

Finally, we call for a renewed focus on methodological rigor and the development of general guidelines and procedures that are applicable across projects and disciplines. More specifically, we need genuine methodological research on CSS, for instance, on how to secure data quality, on how characteristics of citizen scientists shape the knowledge generation process, or on how generalizable and robust CSS research (really) is.
